# Primary care stability as a social determinant

**DOI:** 10.3389/frhs.2025.1646932

**Published:** 2025-08-12

**Authors:** Waseem Jerjes

**Affiliations:** Department of Primary Care and Public Health, Imperial College London, London, United Kingdom

**Keywords:** primary care, health inequalities, workforce stability, social determinants, health policy

## Introduction

Health inequalities in the UK continue from one generation to the next, with those in the most deprived areas experiencing significantly shorter and poorer life years than those in affluent communities (1). The Marmot Review clearly stated that these inequalities do not have to exist, but are the result of unequal access to the social determinants of health—home, education, income, and healthcare itself (2). However, one determinant is consistently neglected: the stability and health of the primary care workforce.

The availability of trusted, continuous, community-based general practice is a powerful lever for health equity. Starfield and colleagues demonstrated that systems anchored in strong primary care deliver better outcomes at lower cost and reduce disparities in morbidity and mortality (3). But access to such care is not evenly distributed. Hart's “inverse care law” remains as relevant today as it was over 50 years ago: the areas with the highest health needs often have the weakest medical provision (4).

The argument of this opinion paper is that stability in the workforce—most critically in general practice—must be acknowledged as an independent social determinant of health. Without a sustainable, supported, and equitably distributed GP workforce, even the best public health strategies will fall short. From burnout to turnover to chronic underinvestment in deprived areas, declining workforce capacity is an acute but slow-moving public health issue. It requires policy recognition not only as a labour market issue, but as an essential cornerstone of fair health systems.

Here, the term “workforce instability” refers specifically to high turnover rates, burnout, and difficulties in retaining primary care professionals, resulting in disrupted continuity of care. Conversely, “workforce maldistribution” describes the uneven geographic spread of clinicians, where the distribution does not match healthcare need—particularly affecting areas with high levels of deprivation.

## Workforce instability as a structural determinant of inequality

The stability and distribution of the core primary care workforce is a structural determinant of health equity, not an organisational issue. Higher deprivation areas tend to have the double burden of greater health need and less access to trained, sustained general practice. This only serves to sustain the inverse care law (5), where practices in deprived areas struggle to recruit, sustain, and provide support for clinicians in the face of greater complexity and administrative pressure.

Evidence repeatedly demonstrates that increased supply of primary care physicians relates to better health outcomes and lower mortality (6). Hospital-centric models may offer equitable access, but without strong general practice foundations, they risk cost inflation, over-medicalisation, and fragmentation (7). Disadvantaged areas, though, experience an uneven spread of these effects. Studies of GP workforces in England reveal that there are fewer GPs per head of population in these areas (8), greater GP turnover (9), and chronic under-recruitment (10).

Importantly, this instability isn't merely about unfilled vacancies—it betrays a fundamental incompatibility between funding, policy, and the reality of frontline care. For example, the existing GP funding allocation largely relies on capitation-based formulae, which inadequately reflect complex patient needs in deprived areas, perpetuating resource shortfalls. Additionally, NHS England's recruitment schemes, such as national incentives aimed broadly rather than specifically targeting deprived localities, have historically failed to significantly reduce GP vacancies in underserved communities.

Salant et al. identified that supply of primary care directly affects the quality of care, and that this gradient of quality tracks closely with social gradients (11). As such, not stabilising the frontlines in high-need areas creates a vicious cycle: overworked GPs, briefer consultations, increased burnout, and less continuity of care—each with disproportionate effects on multimorbid or complex patients.

Such maldistribution must be perceived as a systemic failure, not a choice of the labour force. If no intervention occurs, labour force patterns will simply reflect the structure of deprivation. A sustainable and fair health system cannot exist if the primary health care labour force itself continues to be unevenly supported, deployed, and funded.

At the national policy level, incentive schemes could be introduced, such as financial bonuses, tax benefits, or enhanced professional development funding for primary care practitioners who commit long-term to underserved areas. These policy tools could encourage greater workforce retention and promote a more equitable geographic distribution of healthcare professionals across regions.

In addition to national-level incentives, local compensatory policies tailored to specific economically disadvantaged communities could further enhance stability. These might include supplementary allowances, housing support, and community-specific investments aimed at improving local living and working conditions, thus making these areas more attractive and sustainable for long-term professional commitment.

## Hidden costs: under-resourced practices and unequal burden

Workforce instability in areas of deprivation isn't a discrete issue of HR—it's a chronic failure of policy to invest in proportion to areas of highest need. The practices that service high-deprivation groups tend to have greater multimorbidity, social complexity of need, and administrative burden for relatively fewer resources with which to address the resultant demand (12). This inconsistency, grounded in funding that's structurally inadequate, translates into lower GP morale, increased levels of stress, and feelings of professional futility.

Efforts to address this imbalance must incorporate financial transparency. Analyses of the past have revealed that GP income in poorer areas of deprivation falls short of that in well-off areas, even when patient need has been taken into account (13). Later evidence from NHS England supports that practices who work with these groups of patients work under constrained margins and less infrastructure support (14). This under-resourced environment makes it harder to recruit and retain multidisciplinary teams, compounding pressures on GPs.

Aside from funding, there exists an uneven burden of operation that falls on these practices. They are more liable to experience locum cover difficulties, more susceptible to reporting burnout, and more inclined to describe their caseload as “unmanageable” (15). This partly explains the emotional toll of caring for patients living in poverty, social exclusion, or chronic instability. This movement is an “endless struggle” of those practicing in disadvantaged areas—when complicated multimorbidity is the status quo and biomedical paradigms frequently fail them (16).

In addition, frequent turnover among primary care clinicians negatively impacts the therapeutic relationship between patients and practitioners, eroding trust and making consistent care difficult to achieve. The lack of continuity can also cause significant psychosocial distress for patients, leading to heightened anxiety, diminished feelings of support, and poorer overall health outcomes.

The characterisation of certain patients as “problematic” reflects not their behaviour but the health system that is incapable of accommodating the structure of their lives. Hayes et al. research on “problem patients” reminds us that such a characterisation typically indicates a discord between patient needs and the capacity of the system—not a failure on either side (17).

## From resilience to sustainability: policy levers that shift the burden

General practice in deprived areas has long relied on a narrative of resilience—expecting clinicians to absorb complexity, manage excess demand, and function in under-resourced settings. Yet resilience alone cannot redress structural inequity. Sustainability needs to become the organising philosophy of the workforce in high-need communities, replacing resilience.

Existing funding structures in general practice continue not to address deprivation adequately. Although deprivation measures exist for the Lower-layer Super Output Area (LSOA) level (18), these do not adequately feed into practice-level allocation. The English Index of Deprivation offers rich information on income, housing, education, and health inequalities (19), yet these do not currently influence primary care commissioning sufficiently. Higher deprivation burdens with practices ought to receive funding that is weighted appropriately for the complexity and unmet need they experience, not simply list size.

Together with financing, the performance metrics for primary care do not always measure inequality-sensitive dimensions. Kirkby et al. support the use of advanced summary measures of health inequality in order to guide the design of policies (20). Likewise, Shimonovich et al. demonstrate the better characterisation that can be provided by comparative measures, such as the use of inequality's slope index, of health gaps and that such measures should drive health service investment (21).

Current performance assessments often rely heavily on quantitative indicators, such as patient throughput or consultation volumes, neglecting qualitative aspects of care. A more effective approach would emphasize longitudinal indicators, particularly continuity of care, which better reflect the patient experience, sustained health outcomes, and the stability of clinician-patient relationships.

Beyond the financial supports, working conditions must be addressed in policy. Administrative load, reporting for compliance, and siloed digital systems tend to hit strained teams the worst. When many loads compound, the tipping point for burnout and attrition will quickly be reached, according to Eaton-Hart et al. (22). Professional expectations become overly burdensome in such environments without the safeguard of time for reflection, colleague support, and training.

Policy makers therefore have to introduce place-specific measures: infrastructure funding for impoverished practices, protected time for education and professional development, subsidied mentoring schemes for the locality, and regional locum banks. They are not extravagancies—they are requirements for fair access and good care in the under-provided areas.

Policy measures should also explicitly target continuous professional skills development and technological innovation in grassroots healthcare. These could include structured, government-funded training programmes emphasizing skills tailored to managing complex social determinants of health, and investment in intelligent digital technologies such as telemedicine, AI-driven clinical support tools, and remote monitoring systems to elevate service quality and efficiency in grassroots primary care settings.

## Place-based solutions and training as structural reform

There is a need not only for funding reform but redesigning of the structure of support for, training of, and immersing practitioners in areas of high need. Workforce deficiencies tend to be presented as national crises but become most evident in the locality. Solutions, therefore, have to be tailored to the patterns of deprivation within the locality and synchronised with the everyday realities of those who provide and receive care.

Place-based policies provide one solution. Measures such as funded upgrade of infrastructure, administrative support staff, and localised mentoring programs can alleviate the pressure on practices disproportionally impacted by social complexity. These do, though, take sustained commitment and not short-term pilots. Rivas et al.'s research during COVID-19 demonstrated that GPs in disadvantaged areas suffered greater emotional burden, practice-level stress, and digital exclusion of patients—highlighting how systemic vulnerability is magnified in conditions of crisis (23).

Digital innovations should also be rolled out fairly. As Chappell et al. illustrated, remote consultations rose more in poorer areas during the pandemic, but this wasn't always translated into better access—particularly for older or excluded groups (24). This implies that expanding digitally without community-driven design can widen gaps in care rather than overcome them.

Workforce development must also be rethought. Long-term immersion in underserved environments during GP training has proven to impact subsequent choices of practice location. Palmer et al. discovered that trainees exposed to deprived areas had greater understandings of social determinants, increased clinical flexibility, and better patient engagement skills—but many had no formal support to practice there after training (25). Integration of protected time, supervision, and community career pathways into these placements could translate initial exposure into sustained service.

Effective implementation of place-based reforms can be enhanced significantly through structured partnerships between NHS practices and community organizations. Such collaborations not only improve training by providing practical, community-focused experiences for healthcare professionals, but also build sustainable local networks that directly address community health needs.

These localised measures are not standalone fixes; they are structural reforms that reshape care for historically under-resourced communities. Infrastructure, training, and equity-informed planning have to go hand in hand with one unified vision for primary care transformation.

## Discussion

### Reframing the workforce as prevention infrastructure

The primary care health workers should no longer simply be considered as a mechanism for the provision of health services—this is a type of infrastructure that facilitates or obstructs the overall operation of the health system. Here, the existence of a stable, highly capable, and community-based GP workforce fulfils a preventative purpose as reliably as vaccination, potable water, or secure housing. By the same token, its decay will hurt those who are already suffering social disadvantage most of all.

In spite of ongoing reviews of the workforce and national plans, the UK has not succeeded in halting the attrition trend in high-deprivation areas. Charlesworth et al. identified the ways in how dissatisfaction with work, resource scarcity, and lower levels of income intersect in such environments (26), exacerbating the issues of attraction and retention. However, policy approaches have all too often focused on resilience, adjustment, or short-term reward packages.

What we need, however, is long-term investment in the workforce as a cornerstone of public health. As Richardson et al. contended, inequalities in health result not only from material deprivation but from the way that institutions organise about or ignore marginalised groups (27). If we embrace that primary care saves downstream costs, alleviates multimorbidity, and enables prevention, then not investing in the frontline staff who provide that care isn't simply an organisational failure—it's a driver of inequality.

This perspective aligns with international experiences, such as in Canada and Scandinavian countries, where primary care workforce stability has been increasingly viewed as integral to preventive healthcare infrastructure. For instance, Canada's “Patient's Medical Home” model explicitly emphasizes continuity and workforce retention as core components, resulting in improved population health outcomes, and Scandinavian countries similarly prioritise sustained primary care clinician-patient relationships through supportive policy frameworks.

Investing in this infrastructure equates to viewing GP workforce equity as fundamental to population health planning. This sentiment is echoed by the Lancet Commission on the future NHS workforce in its call for “fit-for-purpose” solutions that link staff with the unmet needs of the population, especially in areas of deprivation (28). This would entail including measures of workforce distribution within national allocation calculations, in combination with deprivation and clinical complexity. The NHS has moved this way with Integrated Care Board allocations (29), but general practice has not yet followed suit.

The value of general practice in preventing illness will only be achieved when the conditions of those who practice it are not an afterthought. To make this shift practical, the following table flags important blind spots in existing workforce policy and outlines an alternative vision for general practice as part of the preventative health infrastructure. This comparative perspective can assist policymakers in framing funding, metrics, and recruitment with population equity in mind (Table 1).

**Table 1:** Workforce stability as a structural determinant in primary care.

Workforce domain	Current policy focus	Equity-relevant blind spot	Reframed role in public health infrastructure
GP numbers	Headcount per capita	Turnover, continuity, local stability	Community health continuity and trust mechanism
Funding formula	Age and disease burden	Social complexity, deprivation load	Enabler of responsive, place-based care
Admin capacity	Underfunded, minimal	Unseen emotional and bureaucratic labour	Shield for clinician bandwidth and safety
Digital innovation	Access targets and platforms	Digital exclusion, usability for vulnerable patients	Connector between system and marginalised populations
Recruitment initiatives	National campaigns	Local retention and peer support	Long-term placement in underserved communities
Performance metrics	Volume and throughput	Continuity, holistic patient outcomes	Metric of community trust and preventive effectiveness

### Innovative directions for policy and practice

Rebuilding the general practice workforce architecture will entail visionary, equity-conscious innovation. A potential direction is to create a General Practice Equity Index that distributes resources not according to population volume, but according to the collective burden on practices—combining measures such as social deprivation, multimorbidity incidence, levels of continuity, and administrative burden. The US already uses the Social Deprivation Index to guide primary care investment with tangible impact on the availability of access and workforce planning, indicating evident global analogues for the English model to emulate (30).

Stabilising care in areas of high deprivation equally requires renewed focus on continuity. Continuity of care is often framed clinically, but it must be reimagined as a structural policy goal. There have been experimentations with cluster trials that showed that financial incentives for sustained GP-patient relationships resulted in tangible gains in the quality of care as well as less fragmentation (31). This model could be made specific to reward practices in the underserved areas for maintaining continuity with patients who tend to experience systemic instability.

Workforce development itself needs to think again. Introducing public health secondments as part of early GP career pathways delivers twin dividends: professional development and greater system understanding. Elsewhere, such placements – with the integration of clinical and community experience – have enhanced retention of the workforce in underserved areas by embedding a place-based responsibility and systemic understanding (32).

Structural sustainability may also be enhanced by federated anchor practices in high-turnover, under-recruitment areas. They would serve as centres of salaried work, mentorship, research, and education. Australia's experience with rural federations shows that this can offer shared infrastructure and diversified pathways of work, and generalism in disadvantaged areas not only becomes possible but desirable (33).

Digital innovation must not serve to entrench exclusion, and so the place of workers with the training to guide patients in the remote software should be situated within the primary care teams. The Scottish mPower programme and US community-based programmes demonstrate the ways that such workers can increase access without burdening clinical staff as well as enabling older and excluded patients to navigate an increasingly digitised system (34).

Lastly, the policy frame must itself change. While many contemporary performance monitoring continues to focus on throughput, new approaches including “Primary Care First” in the US have started to value equity-oriented outcomes: continuity, coordination, and community effects. Implementing these measures across England would refocus general practice on long-term value over short-term volume (35–40). However, these innovative policy proposals carry the risk of unintended adverse outcomes if not robustly backed by sustained political commitment and concrete legislative frameworks. Without such support, initiatives risk becoming fragmented, short-lived, or poorly integrated, potentially exacerbating existing inequities rather than resolving them.

### Policy analysis framework

To systematically evaluate the feasibility and potential impact of the policy interventions proposed in this article, I propose applying a structured policy analysis framework. This framework would classify interventions according to specific criteria such as resource requirements, political feasibility, implementation complexity, and projected impact on health inequalities. Using this structured approach will not only clarify the typology of the interventions but also facilitate discussions around their practicality and scalability in various contexts within the NHS.

For instance, the proposed General Practice Equity Index would be assessed under this framework as a high-impact but politically complex intervention, requiring significant consensus-building and alignment across health authorities. Conversely, place-based mentoring schemes and NHS-community partnerships, while impactful, might represent interventions with moderate resource demands and higher immediate feasibility. Categorising interventions in this manner allows decision-makers to strategically prioritize reforms that balance immediate feasibility against long-term structural benefits.

To further enhance clarity and usability for policymakers, this analysis could be complemented by a comparative graphic summary, such as a policy matrix that visually maps each proposed intervention against estimated impact, complexity, and resource intensity. Such a visual tool would assist stakeholders in rapidly identifying interventions that align with both political priorities and available resources, thus supporting more informed and effective policy decision-making.

### Final reflections: from policy drift to policy resolve

Policy debates about the general practice workforce have languished for too long with inertia—identifying the issue but proposing only fragmentary, short-term solutions. This article has contended that the uneven distribution and volatility of the primary care workforce isn't a service provision concern but a health determinant. The implications are grim: when the public loses stable, ongoing access to general practice, they lose a buffer against deteriorating health and expanding health inequality.

We have to break past rhetorical pledges to equity and start designing policy that treats workforce planning as an instrument for systemic equity. That includes constructing funding models that account for the real cost of complexity, mapping training pathways to areas of maximum priority, and institutionalising responsibility for distribution at all levels of primary care commissioning.

What is being lost is not just trust in individual practitioners, but trust in the system itself. If communities who have historically faced deprivation continue to experience a revolving door of fatigued practitioners, extended waiting lists, and piecemeal care, then confidence in the vision of universal, high-quality care will disappear. In the long term, continued policy inaction risks not only worsening population health outcomes but also eroding public confidence in the capability and fairness of healthcare institutions. Communities repeatedly experiencing inadequate, fragmented primary care are likely to develop lasting mistrust, undermining collective faith in the health system and governmental institutions more broadly. But with political commitment, systemic integrity, and a rethink of the profession as a public good, we can start fixing the fault lines in general practice and make it the cornerstone of an equal NHS.
